# Decreased phase information transfer from the mPFC to the BLA: During exploratory behavior in CUMS rats

**DOI:** 10.3389/fnins.2023.1145721

**Published:** 2023-03-27

**Authors:** Qingying Cao, Zihe Wang, Wenwen Bai, Tiaotiao Liu, Xuyuan Zheng

**Affiliations:** ^1^School of Biomedical Engineering and Technology, Tianjin Medical University, Tianjin, China; ^2^Equipment Department, Tianjin Cancer Hospital Airport Hospital, Tianjin, China

**Keywords:** depression, medial prefrontal cortex (mPFC), basolateral amygdala (BLA), phase information transfer, phase transfer entropy (PTE), exploratory behavior

## Abstract

**Introduction:**

Depression is a mental disorder characterized by aberrant exploratory behavior. Environmental factors, such as chronic stress, are commonly used to induce depression-like behavior in rodent models. The medial prefrontal cortex (mPFC) and the basolateral amygdala (BLA) are crucial sites in subjects with chronic stress-induced depression. The transmission of amplitude information from the mPFC to the BLA was abated during exploratory behavior in depressive rats; however, the nature of the phase interaction between these two sites remains unknown.

**Methods:**

We used chronic unpredictable mild stress (CUMS) to model depression in rats and acquired local field potentials (LFPs) via multiple electrodes implanted in the mPFC and the BLA while rats (both the control and CUMS groups, respectively) were allowed to explore freely in an open field. The weighted phase lag index (WPLI) within the mPFC and the BLA and phase transfer entropy (PTE) from the mPFC to BLA were computed for two groups of rats (control and CUMS rats) to quantify the phase information transmission.

**Results:**

Rats subjected to CUMS showed a decrease in exploratory behavior. The WPLI within the mPFC and the BLA showed strikingly higher phase synchrony at theta frequencies (4–12 Hz) than other frequency bands during exploratory behavior in both the control and CUMS groups. The results of theta PTE from the mPFC to BLA showed that PTE was significantly decreased in the CUMS group compared with the control group.

**Discussions:**

These findings demonstrated that attenuated phase information transfer might restrain exploratory behavior in CUMS rats.

## 1. Introduction

Depression, a common mental disorder affecting an estimated 3.8% of the global population, is a significant contributor to the overall global burden of disease (World Health, 2021). It is characterized by persistent sadness and a lack of interest or pleasure in previously rewarding or enjoyable activities. It can also disturb sleep and appetite. In the worst cases, depression can even lead to suicide. Although depression is a highly heterogeneous syndrome, it is clear that negative stimulation, such as exposure to stress, increases the risk of depression (Ma et al., 2021).

The majority of the literature on depression confirms that one of the main characteristics of depression is a loss of excitatory prefrontal cortical control over the core limbic structures, such as the amygdala, resulting in aberrant processing of rewarding and aversive behaviors (Russo and Nestler, 2013). Accumulating evidence suggests that the medial prefrontal cortex (mPFC) is a key brain region in the regulation of behaviors and emotions (Pfarr et al., 2018; Chen et al., 2021). The basolateral amygdala (BLA), whose aberrations are a major contributor to psychiatric illnesses, such as depression and anxiety, is a pivotal hub that integrates sensory information from the cortical and subcortical areas and drives mood and emotional expression (Munshi and Rosenkranz, 2018; Zheng et al., 2021). A large body of anatomical evidence indicates that structural and functional synaptic changes, including reduced cortical volume, dendritic atrophy, and dendritic spine loss, occur primarily in the mPFC after stress (Gilabert-Juan et al., 2013) while the BLA is inversely altered (Mitra et al., 2005). It is widely accepted that dysregulation of the PFC amygdala drives stress-induced emotional pathology (Hultman et al., 2016). The study by Liu et al. (2020) reveals a dorsal mPFC (dmPFC) to BLA dysregulation based on the connectivity of specific cells with the mPFC in stress-induced anxiety. Another study demonstrated that activation of the mPFC Drd1 pyramidal cells or stimulation of their terminals in the BLA can produce an antidepressant response (Hare et al., 2019). In addition, the mPFC has been shown to suppress depression-induced amygdala-mediated hyperactive affective responses by recruiting BLA inhibitory interneurons (Rosenkranz and Grace, 2001; Bertholomey et al., 2022). Evidence has shown that the activation of Ventromedial prefrontal cortex (vmPFC)–pBLA inputs diminishes chronic unpredictable mild stress- (CUMS-) induced depression- and anxiety-like behaviors *via* activation of the pBLA Calb1 neuron (Yu et al., 2022). These results suggest that mPFC–BLA innervation regulates affective disorders in a BLA inhibition-dependent manner and that depression-like behavior may be associated with a circuit.

Exploration, a crucial component of human and animal behaviors, has been found to be declined in stressed rats (Jacinto et al., 2013; Botta et al., 2020; Dong et al., 2021). The mPFC and the BLA also appear in exploratory behavior. Indeed, it was previously found that stressed rats showed novel exploratory behavioral impairment and that stress affected theta oscillatory coherence within the amygdala-PFC network in the exploration of a novel environment (Jacinto et al., 2013). According to research, increased amplitude synchronization of the mPFC and the BLA in the theta range is related to learned fear and innate anxiety (Likhtik et al., 2014). A previous study reported that information transmission within the mPFC and the BLA predominates the expression of normal emotions (Bao et al., 2021). Subsequently, CUMS rats showed debased connectivity of the mPFC network and reduced information flow from the mPFC to BLA during free exploration (Qi et al., 2020). These outcomes reflect the consequences of amplitude information. In general, it has been suggested that neural oscillations have three basic properties: amplitude, frequency, and phase (Watrous et al., 2015). In contrast to numerous studies focusing on amplitude, phase as a mode of information transfer is largely unstudied for its role in cognition and mood. Philippe used mutual information to organize the different contributions of amplitude, phase, and frequency of oscillation in visual information coding and revealed that phase encoded more visual information than amplitude (Schyns et al., 2011). Phase decoding has the highest accuracy rate among auditory stimulus decoding, indicating that phase contains more abundant task information (Ng et al., 2013). The aforementioned studies reported the importance of phase information transfer in cognitive and emotional tasks, especially in cooperative processing between the brain regions. As previously described, it is central to determining how phase information transmission from the mPFC to the BLA changes during exploratory behavior in depressed rats.

To address this issue, we employed a CUMS-induced rodent depression model. The rats were allowed 10 min of free exploration in an open field, which was the most common test to investigate curiosity, anxiety, and behavior in models of psychiatric disorders (Fonio et al., 2009; Belovicova et al., 2017). Local field potentials (LFPs), which were considered a vital tool to analyse brain network, were obtained synchronously from the mPFC and the BLA in the open field test (OFT; Marceglia et al., 2007). We applied the weighted phase lag index (WPLI) to investigate the interaction between the mPFC and the BLA during exploratory behavior and determined the characteristic frequency of the phase information. Then, we extracted the phase component from LFPs and calculated the phase transfer entropy (PTE) from the mPFC to the BLA in the control and CUMS groups. This study aimed to provide insights into the phase transmission mechanism of the mPFC to BLA circuit in the context of exploratory behavioral disorders for CUMS-induced depression. This might provide a theoretical basis for the alleviation of depressive symptoms through phase-specific oscillatory neuromodulation in the mPFC.

## 2. Materials and methods

### 2.1. Animals

Adult male Sprague–Dawley rats (aged 10–12 weeks, weighing 300–350 g) were used in all experiments. The rats were purchased from the Experimental Animal Center of Tianjin Medical University in China and bred at the animal facility of Tianjin Medical University. The rats were housed on a 12-h light/dark cycle (lights on at 7:00 a.m.) with groups of 3–4 rats per cage at a stable temperature (23°C ± 2°C) and constant humidity (50% ± 5%), except for the CUMS model. All animals had free access to food and water. All procedures were conducted in accordance with the Guide for the Care and Use of Laboratory Animals and the Tianjin Medical University Animal Care and Use Committee (license number: TMUaMEC2021059).

### 2.2. Chronic unpredictable mild stress

According to a previous study (Willner, 1997; Higuchi et al., 2016), a CUMS procedure was performed. Briefly, rats were randomly exposed for 3 weeks to a variety of unpredictable mild stressors: 1-min tail pinch, 5-min cold swimming (4°C), 24-h food deprivation, 24-h water deprivation, inverted light/dark cycle, 12-h stroboscopic light flash (50 Hz flash frequency), 12-h cage tilting, and 12-h soiled bedding. Each stressor was administered once a week. Non-stressed rats were housed under normal conditions.

**Figure 1:**
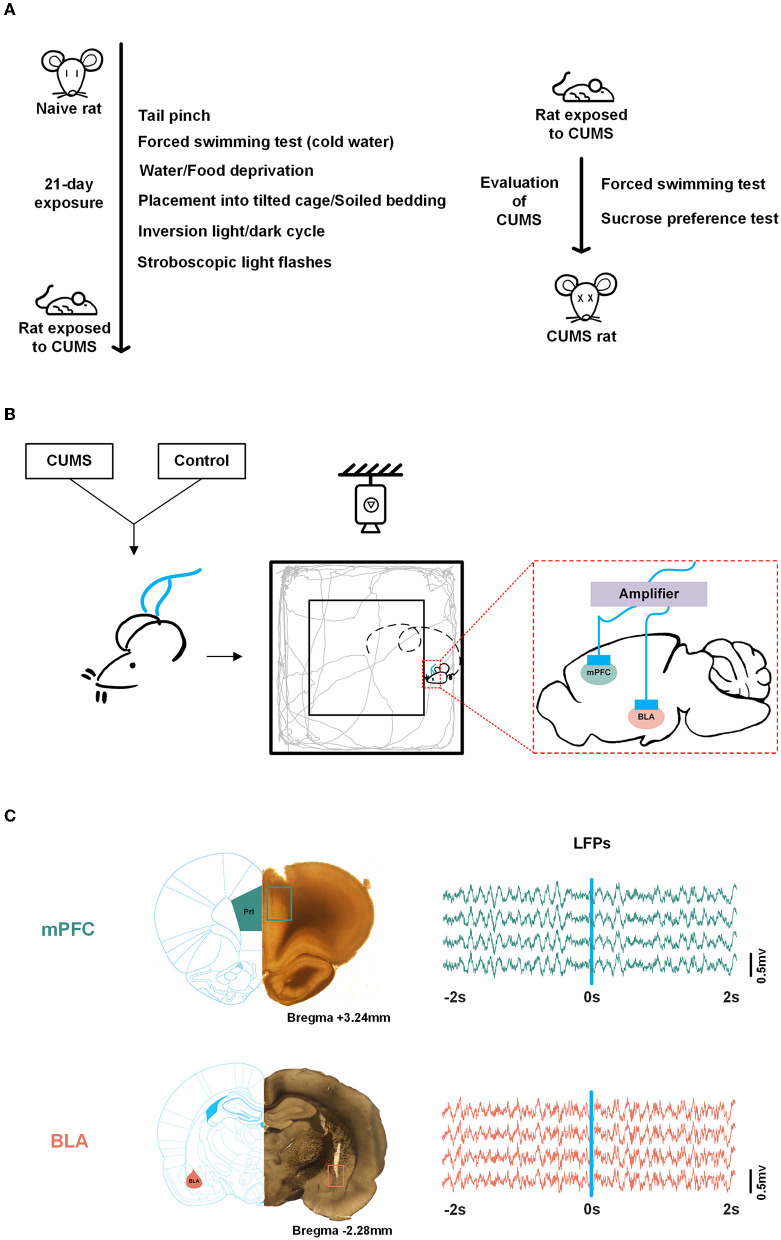
Experimental scheme. **(A)** Scheduling for chronic unpredictable mild stress (CUMS) and validation of the CUMS model. **(B)** Experimental scheme. Both control and CUMS rats were implanted with multichannel microelectrode arrays in the medial prefrontal cortex (mPFC) and basolateral[[Inline Image]] amygdala (BLA). Neural signals were obtained while rats were roaming freely in the open field test (OFT). **(C)** Histological verification of recording sites in the mPFC, PrL region (top, adapted from Wang) and BLA (bottom). Local field potentials (LFPs) data during exploration (−2 to 2 s) in the mPFC (top) and BLA (bottom), respectively. The square refers to the trajectory of the electrode tips. Blue line, reference point (0 s).

The sucrose preference test (SPT) and the forced swimming test (FST) were conducted to validate this model. CUMS-induced depressed rats were successfully prepared by decreasing the sucrose preference rate in the SPT and increasing the immobility time in the FST (Figure 1A).

### 2.3. Open field test

A gray zinc-aluminum alloy chamber (length × width × height, 100 cm × 100 cm × 50 cm) was used as an open field and divided into a central zone (60 cm × 60 cm) and a peripheral zone. During the test, rats were first placed in the center of the field with normal illumination (185 lux) and allowed to explore freely for 10 min. Activities of all rats were recorded using an overhead video monitoring system (Sony, Japan). Center entries, time spent in the central area, rearing times, and the total movement distance were tracked and analyzed using the behavioral analysis software (Ethovision XT8.5, Noldus, The Netherlands). After each test, the open field was scrubbed with 75% ethanol.

When the rats crossed from the periphery to the center, including 2 s in the peripheral zone and 2 s in the central zone, a trial of exploratory behavior was detected and the transition occurred in 0 s.

### 2.4. Surgery and electrophysiological recording

After the successful preparation of these two groups of rats, they were anesthetized with pentobarbital sodium (40 mg/kg, i.p.). They were kept at a temperature of 36°C using a heating pad. Rats were implanted with two multichannel microelectrode arrays (2 × 8 configuration, 50 μm nickel-chromium wires, < 1 MΩ) in the mPFC [+3.5 mm anteroposterior (AP), −0.6 mm mediolateral (ML), and −2.8 mm dorsoventral (DV) from the dura mater] and the BLA (−2.46 mm AP, −4.8 mm ML, and −8.5 mm DV from the dura mater. The aforementioned coordinates were obtained according to a rat brain atlas. The reference electrode was fixed to the skull with screws and dental cement. Postoperatively, in case of incision infection, erythromycin eye ointment and iodophor were applied to the wound suture. Animals were given sustained-release buprenorphine (1 mg/kg) as an analgesic and were allowed to recover for 1 week.

*In* vivo electrophysiological recordings were obtained synchronously from the signal acquisition system during a 10-min open-field exploration in which rats were engaged in activities recorded by overhead video (Figure 1B). Wireline-linked multichannel microelectrode arrays to the neural data acquisition system and neural signals from the mPFC and the BLA were obtained when the OFT was turned on. Wideband neural signals were recorded with a neurophysiological data acquisition system (Plexon, USA). LFPs were amplified (gain: 5,000), bandpass filtered (0.3–120 Hz), and sampled at 2 kHz.

### 2.5. Data analysis

Original LFPs were preprocessed with a 50-Hz notch filter and polynomial fitting to eliminate interference and correct to baseline (Figure 1C). In this study, LFPs were divided into five frequency bands: delta (0.5–4 Hz), theta (4–12 Hz), beta (13–30 Hz), low gamma (30–60 Hz), and high gamma (60–100 Hz). We analyzed the data from each trial. A trial of exploratory behavior was shown as a movement in which the rat crossed from the periphery to the center, including 2 s in the peripheral zone (-2–0 s) and 2 s in the central zone (0–2 s), with the occurrence of the transition in 0 s.

#### 2.5.1. Weighted phase lag index

The weighted phase lag index, a novel measure of phase synchronization, is based on the imaginary cross-spectral component (Vinck et al., 2011). The phase lag index (PLI), proposed by the Stam group, estimated a non-equal probability of phase leads and lags between signals from each brain area, irrespective of the magnitude of phase leads and lags (Stam et al., 2007). However, the discontinuity in this index might hinder the sensitivity of PLI to noise and volume conduction, as small perturbed turn phases lead to phase lags and *vice versa*. The problem is more serious in the case of minor synchronization effects. Compared with PLI, WPLI extends PLI by weighting the contributions of phase leads and phase lags by the magnitude of the imaginary cross-spectral component and consequently alleviating the aforementioned discontinuity. To summarize, we can conclude two main advantages of WPLI: its lower sensitivity to additional noise and its high ability to detect true phase synchronization from real neural data.

To confirm the characteristic frequency band of the phase, we computed the WPLI between the mPFC and the BLA as follows. First, the cross-spectrum was computed using **X** and **Y**, which were transformed from **x** (a signal from the mPFC) and **y** (a signal from the BLA) by the short-time Fourier transform:


(1) $$X_{i}\left(t,f\right)=\int_{-\infty }^{+\infty }\left[x_{i}\left(\tau \right)h\left(\tau -t\right)\right]e^{-j2\pi f\tau }d\tau$$

(2) $$Y_{i}\left(t,f\right)=\int_{-\infty }^{+\infty }\left[y_{i}\left(\tau \right)h\left(\tau -t\right)\right]e^{-j2\pi f\tau }d\tau$$

where **X**_**i**_(**t, f**) and **Y**_**i**_(**t, f**) are short-time Fourier forms of **x**_**i**_(**t**) and **y**_**i**_(**t**) from trial **i** and **h**(**τ − *t***) is the Hamming window with a window length of 250 ms and a moving step of 50 ms, according to the data length from each trial. Moreover, the width of the frequency interval is set as 1 Hz. And, the cross-spectrum is defined as


(3) $$C_{i}\left(t,f\right)=X_{i}\left(t,f\right)Y_{i}^{*}\left(t,f\right),$$

where $Y_{i}^{*}\left(t,f\right)$ indicates the complex conjugate of **Y**_**i**_(**t, f** ).

Then, the WPLI between the mPFC and BLA is calculated as:


(4) $$WPLI=\frac{\left|\sum_{i=1}^{n}ℑ\left(C_{i}\left(t,f\right)\right)\right|}{\sum_{i=1}^{n}\left|ℑ\left(C_{i}\left(t,f\right)\right)\right|},$$

where ℑ(**·**) refers to extracting the imaginary component and **C**_**i**_(**t, f**) is the cross-spectrum, as mentioned in Equation (3).

The weighted phase lag index ranges from 0 to 1. A WPLI of 1 signifies phase locking, where the instantaneous phase of one signal uniformly precedes or lags behind the other signals, while a WPLI of 0 indicates no coupling. If the phase lead or lag of the two signals is random, the WPLI will be close to 0. In conclusion, the higher the phase synchronization, the higher the WPLI.

#### 2.5.2. Phase transfer entropy

Phase transfer entropy, a measure to detect the strength and direction of connectivity between neuronal oscillations, was first presented by Palus and applied to electroencephalogram (EEG) signal analysis by Lobier et al. (2014). In the work referenced earlier, the Lobier group verified that PTE was robust to nuisance factors inherent in the neural signals, such as noise and linear mixing. Its properties are high computational efficiency and a limited number of *a priori* parameters, which reduce computational costs and considerably enhance computational accuracy. A comparison of PTE and real-valued TE (broadband and narrowband TE) showed that PTE is more effective in discovering band-limited directed interactions. Thus, PTE is suitable for estimating directional phase-based connectivity in large-scale investigations. The flow of the phase information between time series extracted from neural signals is quantified by referring to the instantaneous phase sequence as transfer entropy input (Hillebrand et al., 2016).

First, a zero-phase-shift digital filter was used to obtain the specific frequency LFPs, and their instantaneous phase series was computed by the Hilbert transform as follows:


(5) $$H\left(S\left(t\right)\right)=\frac{1}{\pi }\int_{-\infty }^{+\infty }\frac{S\left(\tau \right)}{t-\tau }d\tau$$

(6) $$\theta \left(t\right)=arctan\frac{H\left(S\left(t\right)\right)}{S\left(t\right)}$$

where **S**(**t**) represents the filtered LSPs for a given frequency, **H**(**S**(**t**)) is the Hilbert transform of **S**(**t**), and **θ**(**t**) is the instantaneous phase of **S**(**t** ).

Then, we define PTE from channels **i** to **j** with a given time lag **δ** as:


(7) $$\begin{array}{l} PTE_{ij}=SH\left(\theta_{j}\left(t\right),\theta_{j}\left(t-\delta \right)\right)+SH\left(\theta_{j}\left(t-\delta \right),\theta_{i}\left(t-\delta \right)\right)\\ \;-SH\left(\theta_{j}\left(t\right),\theta_{j}\left(t-\delta \right),\theta_{i}\left(t-\delta \right)\right)-SH\left(\theta_{j}\left(t-\delta \right)\right) \end{array}$$

where **SH**(**·**) indicates the Shannon entropy, **θ**(**t**) indicates the instantaneous phase, and **t** is the time. **δ** refers to the time lag coefficient (**δ = 10 ms**). The formula can be expanded as:


(8) $$\begin{array}{l} \;PTE_{ij}=\sum p\left(\theta_{j}\left(t\right),\theta_{j}\left(t-\delta \right),\theta_{i}\left(t-\delta \right)\right)\\ \;\log_{2}\left(\frac{p\left(\theta_{j}\left(t\right),\theta_{j}\left(t-\delta \right),\theta_{i}\left(t-\delta \right)\right)p\left(\theta_{j}\left(t-\delta \right)\right)}{p\left(\theta_{j}\left(t\right),\theta_{j}\left(t-\delta \right)\right)p\left(\theta_{j}\left(t-\delta \right),\theta_{i}\left(t-\delta \right)\right)}\right), \end{array}$$

where **p**(**·**) denotes probability.

A phase-space binning method is used to calculate joint marginal distributions of signals, as mentioned in Eq. (8). A single histogram-based probability function is built by appropriately binning the occurrence of a single, pair of, or three instantaneous phases in each trial. Bin width is defined based on Scott's choice (Scott, 1992):


(9) $$w\left(a\right)=\frac{3.5\sigma \left(a\right)}{N^{\frac{1}{3}}},$$

where **w(*a*)** denotes the bin width for phase time-series (**a = θ**_**j**_, **θ**_**j**_(**t** − δ) **or** θ_**i**_(**t** − δ)), **σ**(**a**) the standard deviation (SD) of **a**, and **N** the number of **a**. Hence, the number of bins is: $\text{k}\left(\text{a}\right)=\frac{2\pi }{\text{w}\left(\text{a}\right)}$ and PTE is further represented as:


(10) $$\begin{array}{l} PTE_{ij}=\sum_{k_{1}=1}^{k\left(\theta_{j}\left(t\right)\right)}\sum_{k_{2}=1}^{k\left(\theta_{j}\left(t-\delta \right)\right)}\sum_{k_{3}=1}^{k\left(\theta_{i}\left(t-\delta \right)\right)}\frac{N\left(k_{1},k_{2},k_{3}\right)}{N}\log2\\ \;\frac{N\left(k_{1},k_{2},k_{3}\right)N\left(k_{2}\right)}{N\left(k_{1},k_{2}\right)N\left(k_{2},k_{3}\right)} \end{array}$$

Finally, the mean PTE from the mPFC to BLA is defined as:


(11) $$PTE=\frac{\sum_{i=1}^{N_{m}}\sum_{j=1}^{N_{b}}PTE_{ij}}{N_{m}\times N_{b}},$$

where **N**_**m**_ and **N**_**b**_ are channel numbers from the mPFC and the BLA, respectively.

Here, we considered an approach to evaluate the statistical significance of each PTE value. We shuffled the time series from each trial and used the bootstrapped data to compute PTE to obtain a curve of the proxy PTE. This procedure was repeated 500 times for each trial. Then, we generated a distribution of peak PTE of the shuffled controls from one trial. The 0.95 percentile of the mean peak PTE of shuffled controls becomes the significance level. The significance of connections is assessed by comparing the actual against the mean and SD of the mean shuffled controls, which allows for further confidence in the measure of estimated causality.

### 2.6. Histology

After all experiments, the rats were deeply anesthetized and perfused with phosphate-buffered saline (PBS) and 4% paraformaldehyde. Brain sections were obtained with a vibratome (Vibratome, USA). Recording sites were observed under an optical microscope (Olympus, Japan) and verified on a rat brain atlas.

### 2.7. Statistical analysis

All data were expressed as mean ± standard error of the mean (SEM). Methods and the sample size for statistics are described in the figure legend. Data were analyzed *via* Student's *t*-test (the Wilcoxon matched-pair signed-rank test and the Mann–Whitney test for a comparison of two groups) and one- or two-way repeated measures analysis of variance (ANOVA) followed by *post hoc* multiple comparisons with Dunnett's and Bonferroni's test, respectively. Circular statistics, such as the Watson-Williams test, were also gathered to assess the significance of the phase distribution. Pearson's correlation was applied to determine the correlation between WPLI/PTE and the motion of rats. The statistical significance threshold was set at a *p*-value of < 0.05.

## 3. Results

### 3.1. CUMS induces depressive-like behaviors during OFT

Chronic unpredictable mild stress is a commonly used rodent model of depression; hence, we performed a modified CUMS paradigm to induce a model of depression in rats. After 3 weeks of CUMS treatment, the rats showed typical depressive-like phenotypes, as evaluated by the SPT and the FST (Supplementary Figure 1).

**Figure 2:**
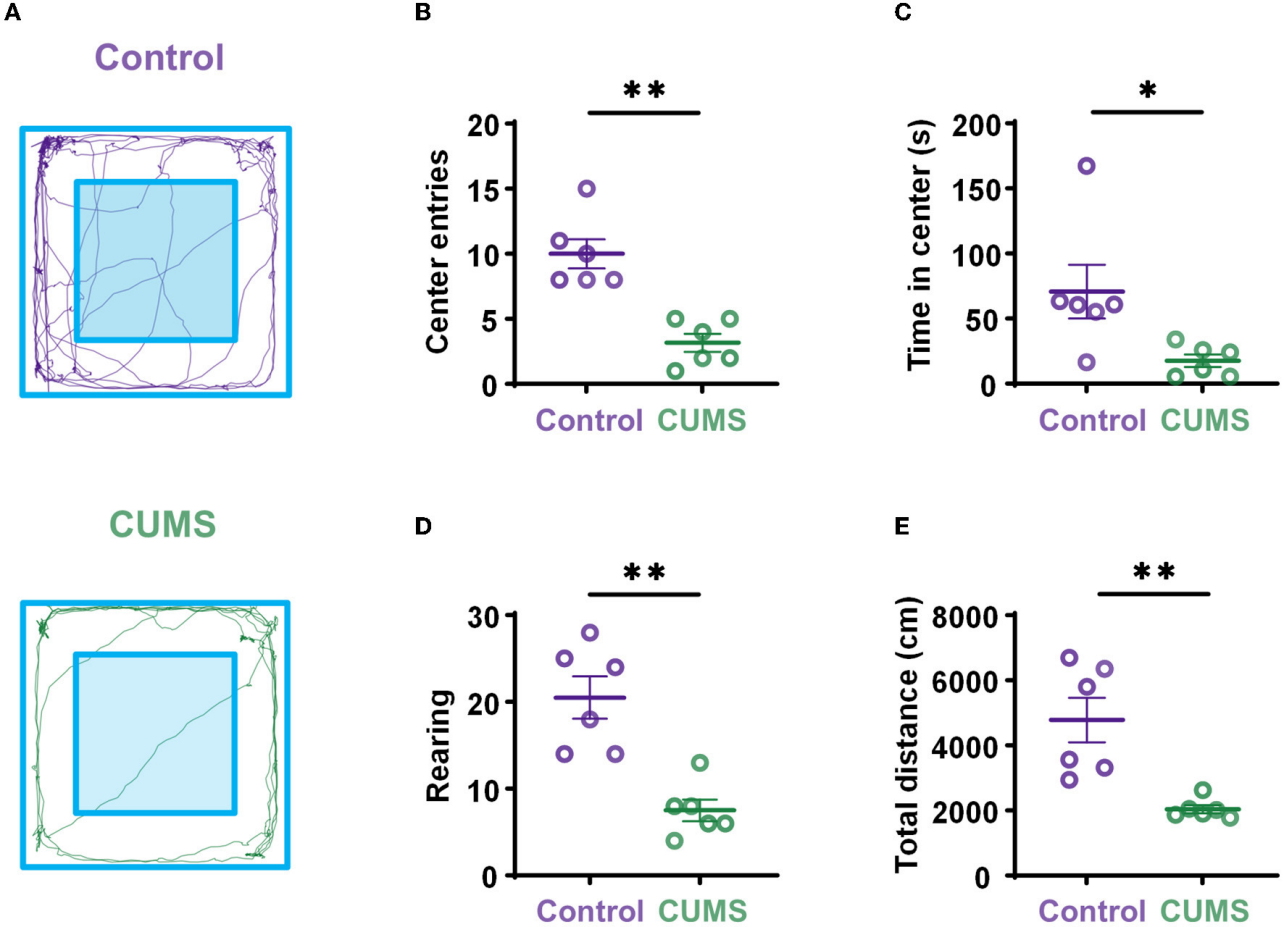
Depressive-like behaviors induced by exposure to CUMS. **(A)** Representation of open-field exploration trajectories in the control (top) and CUMS groups (bottom). **(B)** Center entries during the OFT. **(C)** Time in center during the OFT. **(D)** Rearing times during the OFT. **(E)** Total movement distance during the OFT. Error bars indicate standard error of the mean (SEM). Two-tailed Mann–Whitney test, *p < 0.05, **p < 0.01.

To identify the effects of prolonged CUMS on exploratory behavior, rats underwent a 10-min OFT. Although rodents showed thigmotaxis, CUMS rats still showed little motor activity, more grooming, and a low level of rearing (Figure 2A). The results showed that the behavioral parameters of CUMS rats in the OFT, including center entries (10 ± 1.125, *n* = 6 rats for control vs. 3.167 ± 0.703, *n* = 6 rats for CUMS, *p* < 0.01, Figure 2B), time in the center area (70.658 ± 20.610, *n* = 6 rats for control vs. 17.639 ± 4.886, *n* = 6 rats for CUMS, *p* < 0.05, Figure 2C), rearing times (20.5 ± 2.466, *n* = 6 rats for control vs. 7.5 ± 1.258, *n* = 6 rats for CUMS, *p* < 0.01, Figure 2D), and total movement distance (4,780.653 ± 687.37, *n* = 6 rats for control vs. 2,040.826 ± 123.613, *n* = 6 rats for CUMS, *p* < 0.01, Figure 2E), were significantly lower than those of control rats, indicating that CUMS could induce depressive-like behaviors and decrease willingness to explore in the open field.

### 3.2. Phase synchronization between the mPFC and the BLA in theta-frequency augments during exploratory behavior

**Figure 3:**
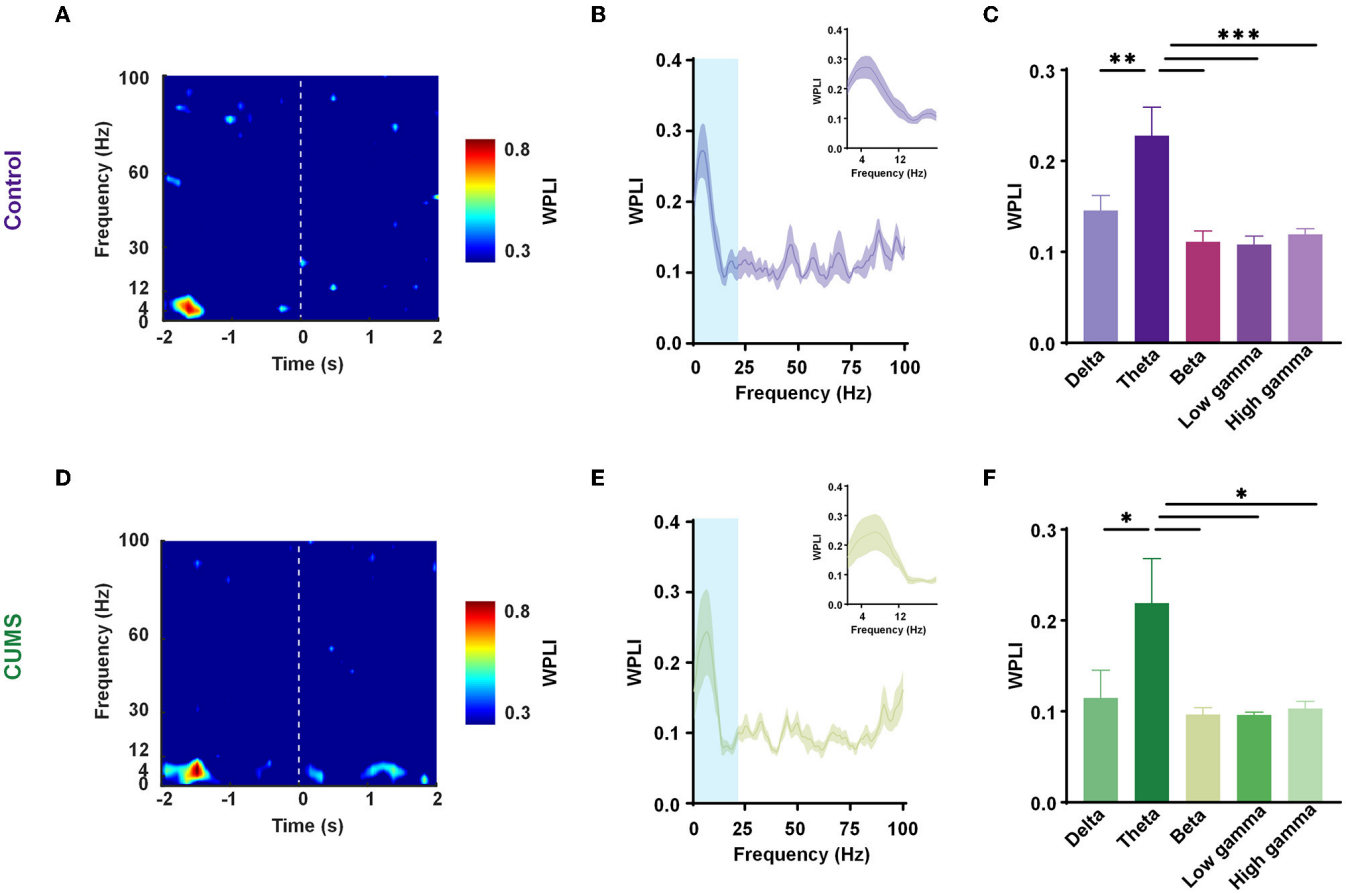
Enhanced mPFC–BLA phase synchrony in the theta range before entering into the center. **(A, D)** Representative spectrograms of the weighted phase lag index (WPLI) during exploration [transition from the periphery (−2 to 0 s) to the center (0–2 s)] in the control **(A)** and CUMS groups **(D)**. Dashed line, reference point (0 s). **(B, E)** The mean WPLI curve with a frequency in the periphery for the control **(B)** and CUMS groups **(E)**. Inset zooms in on the light blue area. *n* = 6 rats per group. Faded bands, ±SEM. **(C, F)** WPLI of the control **(C)** and CUMS groups **(F)** in different frequency bands. *n* = 6 rats per group. Error bars indicate SEM. One-way analysis of variance (ANOVA), *p < 0.05, **p < 0.01, ***p < 0.001.

Previous research has shown an increase in theta power in the mPFC and the BLA during a transition from the periphery to the center (Likhtik et al., 2014). The cooperative long-scale information processing could be well reflected in the phase of neural oscillations. Hence, our analysis focused on the phase component of LFPs in the two sites. In trials of exploratory behavior, WPLI with better noise robustness and less volume conduction interference was calculated at all frequencies. According to the aforementioned study, exploratory behavior was defined as the movement of rats going into the central zone from the peripheral zone, which contained 2 s in the center and 2 s in the periphery. The time-frequency diagram showed a prominent theta frequency component at approximately −1 s (where rats were in the peripheral area of the open field; Figures 3A, D; Supplementary Figure 2). The curves of WPLI over frequency in the two groups showed significantly higher phase synchronization within the mPFC–BLA circuit in the theta-frequency transition from the periphery to the center (Figures 3B, E). A one-way ANOVA revealed a main effect for frequencies in the two groups [*F*_(4,25)_ = 8.223, *p* < 0.001, *n* = 6 rats 130 trials for control; *F*_(4,25)_ = 3.981, *p* < 0.05, *n* = 6 rats; 130 trials for CUMS], and Dunnett's multiple comparisons test demonstrated that theta frequency had a statistically significant difference as compared to other frequency bands (theta vs. delta: *p* < 0.01, theta vs. beta: *p* < 0.001, theta vs. low gamma: *p* < 0.001, theta vs. high gamma: *p* < 0.001 for control; theta vs. delta: *p* < 0.05, theta vs. beta: *p* < 0.05, theta vs. low gamma: *p* < 0.05, and theta vs. high gamma: *p* < 0.05 for CUMS; Figures 3C, F). Interestingly, when CUMS and control rats were in the peripheral area of the open field, the mPFC–BLA theta-phase synchrony was enhanced.

**Figure 4:**
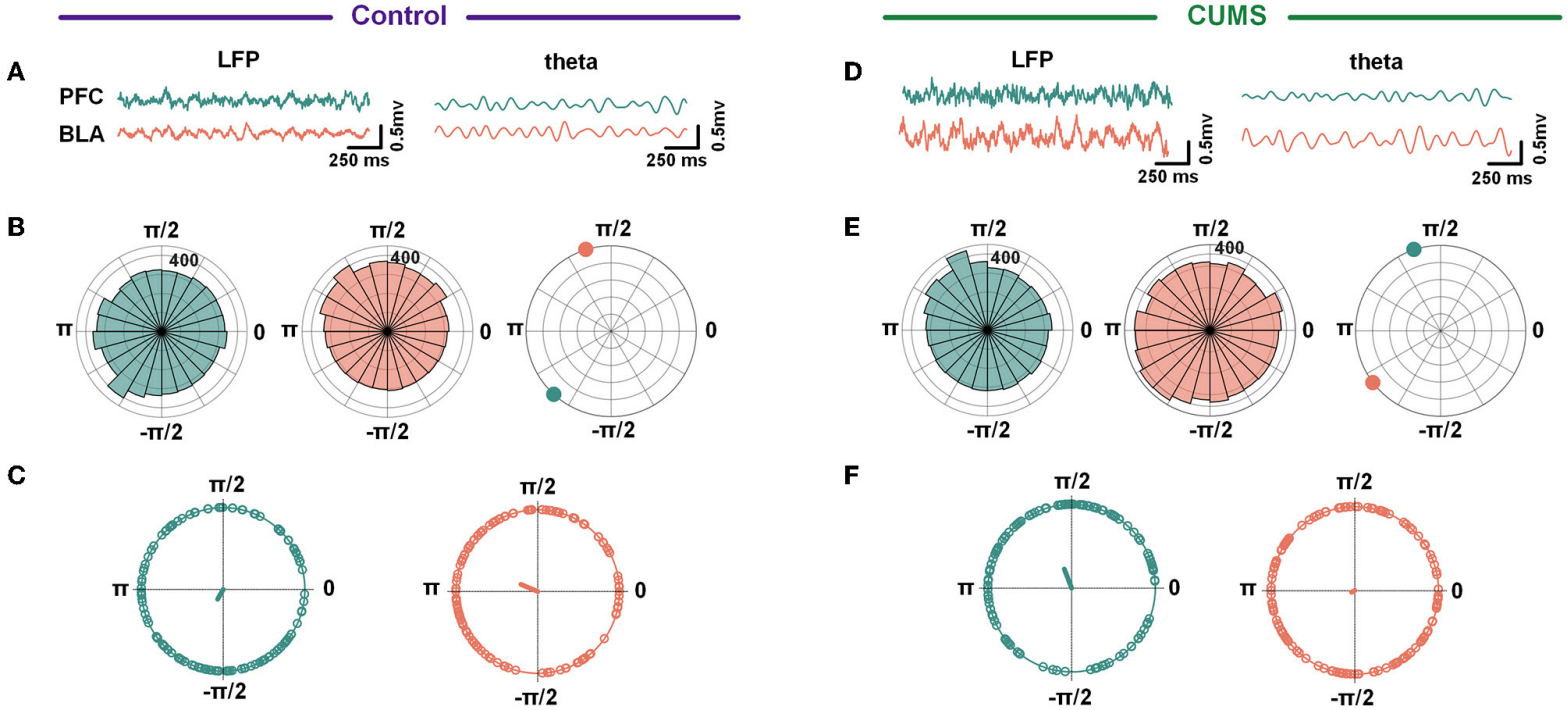
A circular histogram of theta frequency phase. **(A, D)** Example of raw LFP (left) and theta band filtered trace (right) from the mPFC and BLA in the control **(A)** and CUMS groups **(D)**. **(B, E)** A circular histogram of the theta phase in the mPFC (green) and BLA (red) in the control **(B)** and CUMS groups **(E)**. **(C, F)** The phases of theta in the mPFC were significant with the phases of theta in the BLA for both control **(C)** and CUMS groups **(F)**. Control group: 240.43° = −2.09 for the mPFC, 157.06° = 2.74 for the BLA, Watson-Williams test, *F* = 27.19, *p* < 0.001; CUMS group: 111.14° = 1.94 for the mPFC, 208.45° = −2.65 for BLA, Watson-Williams test, *F* = 16.04, *p* < 0.001. Green/red dots depict individual trials, *n* = 6 rats 121 trials for the control group, *n* = 6 rats 99 trials for the CUMS group.

In addition, in a two-way repeated-measures ANOVA, there was no main effect for groups [*F*_(1,50)_ = 1.360, *p* > 0.05], no significant interaction effect [*F*_(4,50)_ = 0.071, *p* > 0.05], and an inverse main effect for frequency [*F*_(4,50)_ = 10.48, *p* < 0.001; Supplementary Figure 3]. The result showed a decreasing trend in WPLI with no statistically significant difference between the CUMS and the control group. Despite no change in overall synchronicity, the preferred phase distribution of the theta-range in the mPFC and the BLA of CUMS and control rats changed markedly (CUMS vs. control: *F* = 60.12, *p* < 0.001 for the mPFC; CUMS vs. control: *F* = 5.14, *p* < 0.05 for the BLA; Watson-Williams test; Figure 4). The results abovementioned indicated that theta-frequency might be related to the aberrant phase interaction within the mPFC–BLA between the control and CUMS groups.

### 3.3. Phase transfer information from the mPFC to BLA attenuates in open-field exploration in CUMS rats

**Figure 5:**
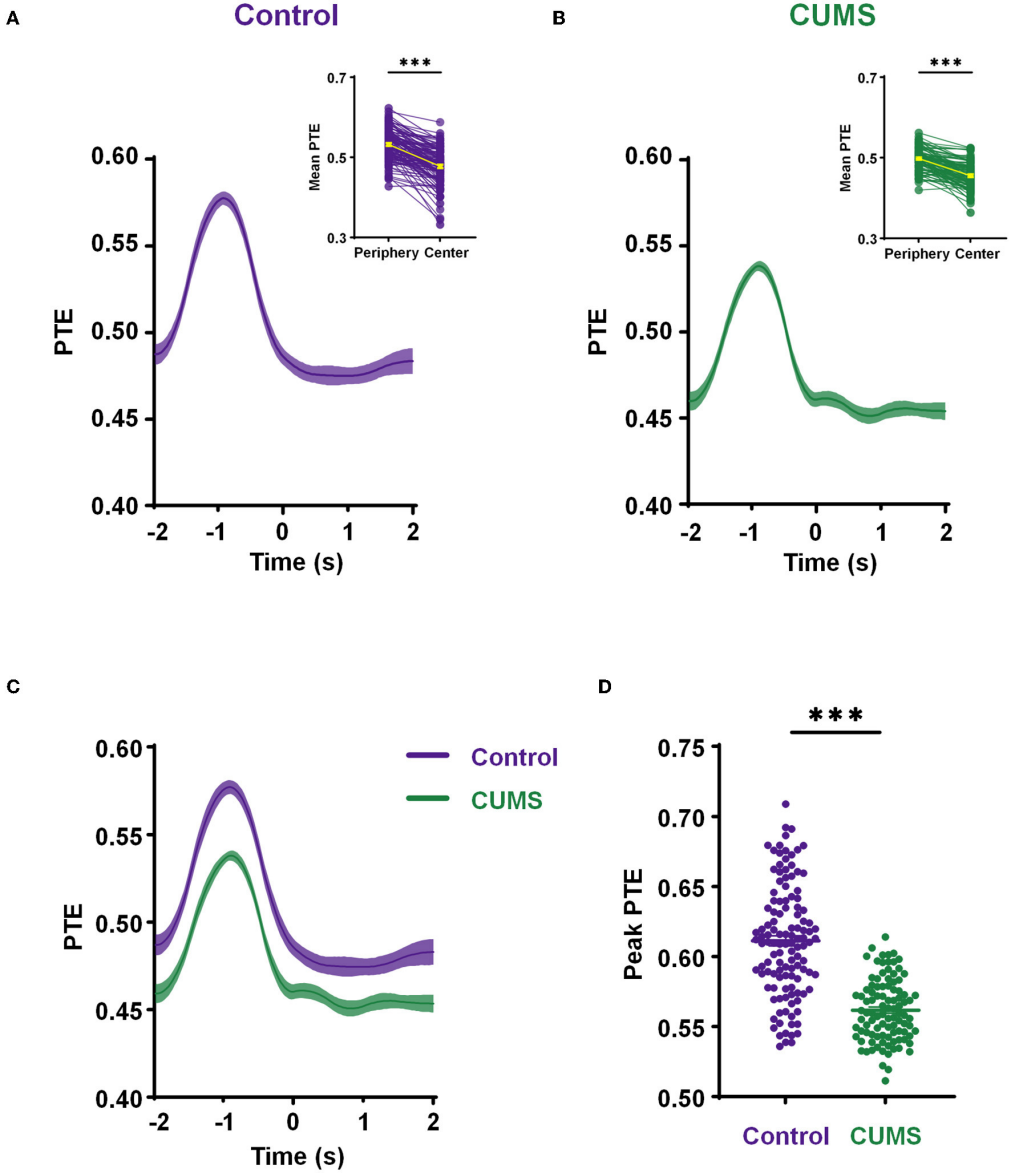
Decreased phase information transmission in the theta range from the mPFC to BLA during exploration. **(A, B)** The mean phase transfer entropy (PTE) (from the mPFC to BLA) curve with time in theta-frequency during exploratory behavior of the control **(A)** and CUMS groups **(B)**. Inset shows a comparison of the mean PTE in the periphery (−2 to 0 s) and center (0–2 s), a yellow line indicates the mean value. Wilcoxon matched pairs signed rank test, ***p < 0.001. **(C)** A comparative diagram of the mean PTE in the control and CUMS groups. **(D)** The comparison of theta-band peak PTE from the mPFC to BLA between the control and CUMS groups. Two-tailed Mann–Whitney test, ***p < 0.001. *n* = 6 rats 121 trials for the control group, *n* = 6 rats 99 rats for the CUMS group. Faded bands, ±SEM. Dots, individual trials.

Given the importance of theta-frequency phase oscillations in long-range synchronization within the mPFC–BLA circuit during exploratory behavior, we examined how phase information transfer changed. Therefore, we calculated PTE at the theta-frequency from the mPFC to the BLA during an exploration of 4 s, including 2 s in the periphery and 2 s in the center. As rats (both CUMS and control rats) moved from the periphery to the center, PTE in the theta-range elevated to a peak at ~1 s before transitioning into the center and then gradually reduced back to the baseline (control: 0.533 ± 0.004, *n* = 6 rats 121 trials for the periphery vs. 0.478 ± 0.004, *n* = 6 rats 121 trials for the center, *p* < 0.001; CUMS: 0.498 ± 0.003, *n* = 6 rats 99 trials for the periphery vs. 0.455 ± 0.003, *n* = 6 rats 99 trials for the center, *p* < 0.001; Wilcoxon test; Figures 5A, B; Supplementary Figure 4). It reveals a phase information transfer characteristic, in that the theta-PTE from the mPFC to the BLA increases when rats are in the periphery of the open field.

To further investigate these relationships, the mean theta-PTE from the mPFC to the BLA for a comparison of CUMS and control rats was displayed. This reflected a remarkably lower PTE in rats exposed to CUMS than that in control rats (Figure 5C). Subsequently, an independent *t*-test showed that the peak of theta-PTE in the CUMS group was significantly reduced compared to the control group (0.611 ± 0.004 *n* = 6 rats 121 trials for control vs. 0.562 ± 0.002 *n* = 6 rats 99 trials for CUMS, *p* < 0.001; Mann–Whitney test; Figure 5D). These results imply that CUMS reduces the phase transfer from the mPFC to the BLA when rats traverse from the periphery to the center of the open field. All data from the abovementioned PTE analysis above were significantly higher than the values obtained from shuffled controls (Supplementary Figure 5). The connections of PTE from the mPFC to the BLA were demonstrated to be real, and the values of PTE were related to exploratory behavior.

Altogether, phase plays a crucial role in CUMS-induced depressive-like behaviors, such as exploratory hypoactivity. Abated phase information transfer of mPFC–BLA innervation may result in reduced exploratory behavior in CUMS rats.

### 3.4. Correlation

**Figure 6:**
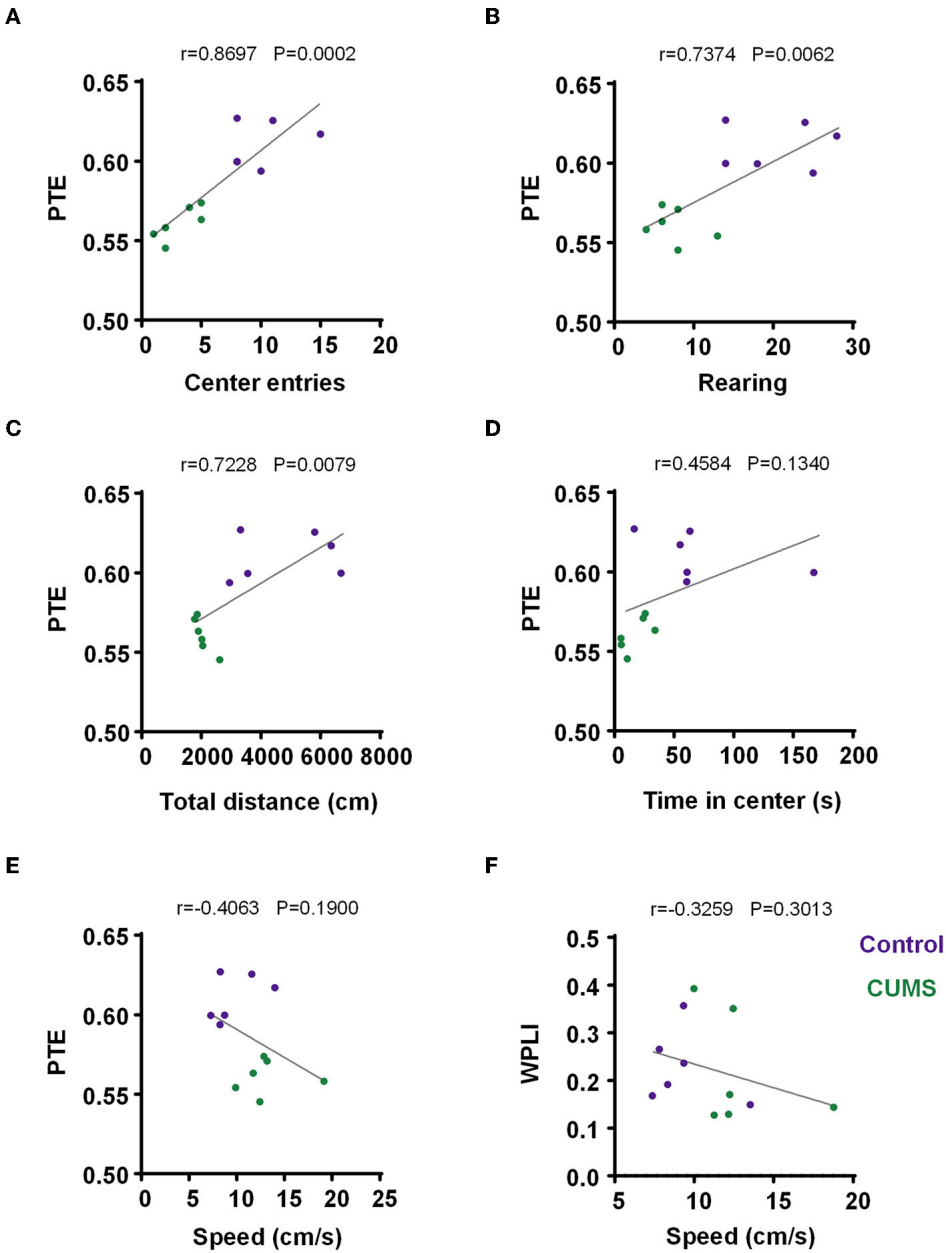
Correlation between PTE/WPLI and rodent behavior. **(A)** Correlation between the PTE and center entries for the control and CUMS groups. **(B)** Correlation between PTE and rearing times for the control and CUMS groups. **(C)** Correlation between PTE and total movement distance in the OFT for the control and CUMS groups. **(D)** Correlation between PTE and time in the center for the control and CUMS groups. **(E)** Correlation between PTE and motion velocity in an open field for the control and CUMS groups. **(F)** Correlation between WPLI and motion velocity in the open field for the control and CUMS groups. **(A–F)** Dots depict each rat, *n* = 6 rats per group. Linear regression analysis and Pearson's correlation were performed.

For depressive-like phenotypes, there were positive and significant correlations between PTE and behaviors (center entries: *r* = 0.8697, *p* = 0.0002, *n* = 6 rats per group, Figure 6A; rearing times: *r* = 0.7374, *p* = 0.0062, *n* = 6 rats per group, Figure 6B; total movement distance in the OFT: *r* = 0.7228, *p* = 0.0079, *n* = 6 rats per group, Figure 6C). Additionally, an analysis of PTE and time in the center did not have a significant correlation (*r* = 0.4584, *p* = 0.1340, *n* = 6 rats per group, Figure 6D). The results implied that decreased PTE from the mPFC to the BLA in CUMS rats was behaviorally relevant.

In both CUMS and control rats, no correlation was observed between the movement speed and WPLI/PTE of each subject (PTE: *r* = −0.4063, *p* = 0.1900, *n* = 6 rats per group, Figure 6E; WPLI: *r* = −0.3259, *p* = 0.3013, *n* = 6 rats per group, Figure 6F). Briefly, the movement of animals had no impact on task-induced changes in WPLI and PTE.

## 4. Discussion

Consistent with the current research that a CUMS model could produce depressive-like behaviors, experiments conducted in this study revealed that CUMS reduced the free exploration of rats in the OFT. Importantly, we analyzed the phase interaction of long-scale brain regions in exploratory behavior and found a significantly increased phase synchronization in the theta range within the mPFC–BLA when rats were in the open-field peripheral area prior to the center. Moreover, phase information transmission was quantified by PTE. The result showed that the theta band phase transmission from the mPFC to the BLA was notably higher at the periphery than at the center of the open field. Also, in the CUMS group, it was observed that phase information transfer of theta frequency from the mPFC to the BLA during exploratory behavior was attenuated compared to the control group. Together, this study revealed an impairment of the information transmission mechanism from the mPFC to the BLA from the perspective of phase underlying the impediment of CUMS-induced exploratory behavior, that is, a weakening of phase transfer, which we predicted to result in the disturbance of exploration in CUMS rats. The debased phase information transfer along with a decline in exploratory behavior in CUMS-induced depressive-like rats might aid in monitoring the state of depression.

The medial prefrontal cortex is a pivotal target of stress and chronic stress-induced psychiatric disorders containing depression (Arnsten, 2009, 2015). Specifically, it has extensive connections to the amygdala for the regulation of aberrant emotions (Senn et al., 2014; Zhang et al., 2019). Projections from the mPFC to the medial BLA are involved in the top-down control of emotional behaviors, and PFC can drive the firing of neurons in the BLA (McGarry and Carter, 2017). Liu's study suggested that chronic stress shifted the excitatory-inhibitory balance in the Dorsomedial prefrontal cortex (dmPFC) to BLA projection neurons (PNs) due to enhanced presynaptic glutamate release (Liu et al., 2020). Moreover, prolonged severe stress induces attenuated glutamatergic projection from pyramidal neurons in the PFC to GABAergic interneurons in the BLA, resulting in the inhibition barrier from the PFC and ensuring hyperexcitability of BLA PNs, which causes behavioral disorders (Wei et al., 2017). Our finding demonstrated that decreased phase information transfer might embody dysregulated projections from the PFC to the BLA.

In addition, the diminished flow of amplitude information from the mPFC to the BLA leads to an impairment of exploratory behavior in CUMS rats (Qi et al., 2020). Decreased phase-amplitude coupling between the mPFC and the BLA may inhibit exploratory behavior in CUMS-induced depressive rats (Wang et al., 2021). These findings emphasize the role of the amplitude of neural oscillations in CUMS-induced depressive-like behaviors. Thus, our subsequent analysis quantifies the transmission of amplitude information from the mPFC to the BLA *via* amplitude transfer entropy similar to PTE and compares it quantitatively with phase transfer.

Additionally, the study suggests that there is a closed-loop communication between the PFC and BLA with reciprocal interactions in both directions (McGarry and Carter, 2016). Projections from the BLA to mPFC are also directly involved in the regulation of anxiety-related behavior, and the Corticotrophin-releasing factor 1 (CRF1) receptors in mPFC layer V pyramidal cells, in which CRF1-inputs are majorly from BLA, are activated by exposure to stress (Liu et al., 2015). It was also inferred that stress enhances a functional coupling in BLA–mPFC, the activation of which is anxiogenic and generates negative affective states (Marcus et al., 2020). Another study revealed that the BLA–PFC circuit is critical in the prediction of rewards and punishment (Burgos-Robles et al., 2017). These prevailing views implied that the information transfer between the mPFC and BLA might be unidirectional or reciprocal. Therefore, it is essential for future studies to investigate the orientation of particular phase information between the mPFC and BLA in exploration after exposure to chronic stress.

Mental disorders usually involve disruption of cognitive functions, such as emotional dysregulation, impaired decision-making, or abnormal behaviors. Mental illnesses can be diagnosed and treated by identifying and restoring abnormalities in network activity to restore health because network activity generates those cognitive functions as a carefully timed and orchestrated synchronous concrete manifestation in a large-scale structure (Kanta et al., 2019). Brain stimulation is a good way to restore healthy network activity. Accumulating transcendental knowledge has shown that optogenetic stimulation of PFC-IL produces a rapid and long-lasting antidepressant effect by enhancing the number and function of spine synapses of layer V pyramidal neurons in the PFC (Fuchikami et al., 2015). Another study proved that photostimulation of Drd1 neurons in the PFC produces rapid antidepressant responses that aid in probing cellular target neurons (Hare et al., 2019). The chemogenetic inhibition of GABA interneurons in the mPFC indicates a sufficient way for antidepression, implying that the inhibition of Gamma - aminobutyric acid (GABA) interneurons facilitates synaptic plasticity underlying rapid antidepressant effects (Fogaca et al., 2021). Meanwhile, it was observed that precise optogenetic stimulation of BLA terminals in the central nucleus of the amygdala exerts an anxiolytic effect not only by simply targeting specific cells but also by targeting defined circuit projections (Tye et al., 2011). However, both invasive and non-invasive neuromodulation has yielded inconsistent results. It was argued that existing neuromodulation therapies have only spatial specificity, deliver energy to the specific areas of the brain without temporal specificity, and fail to transmit energy consisting of intrinsic brain activity (Widge and Miller, 2019). Thus, the remediation of a particular brain function would be best achieved by stimulating precisely at specific phases of a particular oscillation. Phase-dependent stimulation has also been shown to be conducive to treating neurological and psychiatric disorders (McNamara et al., 2020). This finding could contribute to a more refined strategy to modulate theta oscillations in the treatment of phase-based depression. According to such methods, stimulation that is in phase with a rhythm can boost network activity, while stimulation that is out of phase can diminish it. The application of phase-dependent stimulation to antidepressant responses could be ulteriorly demonstrated in our next work using optogenetic stimulation targeting specific populations of neurons.

## Data availability statement

The original contributions presented in the study are included in the article/Supplementary material, further inquiries can be directed to the corresponding authors.

## Ethics statement

The animal study was reviewed and approved by Tianjin Medical University Animal Care and Use Committee (license number: TMUaMEC2021059).

## Author contributions

XZ and TL designed the experiment. QC, ZW, and WB carried out the experiment. QC and TL analyzed the data. QC and XZ wrote the manuscript. All authors read and approved the final manuscript.

## References

[B1] Arnsten A. F. (2009). Stress signalling pathways that impair prefrontal cortex structure and function. Nat. Rev. Neurosci. 10, 410–422. 10.1038/nrn2648 19455173 PMC2907136

[B2] Arnsten A. F. (2015). Stress weakens prefrontal networks: molecular insults to higher cognition. Nat. Neurosci. 18, 1376–1385. 10.1038/nn.4087 26404712 PMC4816215

[B3] Bao X. Qi C. Liu T. Zheng X. (2021). Information transmission in mPFC-BLA network during exploratory behavior in the open field. Behav. Brain Res. 414, 113483. 10.1016/j.bbr.2021.113483 34302874

[B4] Belovicova K. Bogi E. Csatlosova K. Dubovicky M. (2017). Animal tests for anxiety-like and depression-like behavior in rats. Interdiscip. Toxicol. 10, 40–43. 10.1515/intox-2017-0006 30123035 PMC6096862

[B5] Bertholomey M. L. Nagarajan V. Smith D. M. Torregrossa M. M. (2022). Sex- and age-dependent effects of chronic corticosterone exposure on depressive-like, anxiety-like, and fear-related behavior: Role of amygdala glutamate receptors in the rat. Front. Behav. Neurosci. 16, 950000. 10.3389/fnbeh.2022.950000 36212195 PMC9537815

[B6] Botta P. Fushiki A. Vicente A. M. Hammond L. A. Mosberger A. C. Gerfen C. R.. (2020). An amygdala circuit mediates experience-dependent momentary arrests during exploration. Cell. 183, 605–619.e622. 10.1016/j.cell.2020.09.023 33031743 PMC8276519

[B7] Burgos-Robles A. Kimchi E. Y. Izadmehr E. M. Porzenheim M. J. Ramos-Guasp W. A. Nieh E. H.. (2017). Amygdala inputs to prefrontal cortex guide behavior amid conflicting cues of reward and punishment. Nat. Neurosci. 20, 824–835. 10.1038/nn.4553 28436980 PMC5448704

[B8] Chen Y. H. Wu J. L. Hu N. Y. Zhuang J. P. Li W. P. Zhang S. R.. (2021). Distinct projections from the infralimbic cortex exert opposing effects in modulating anxiety and fear. J. Clin. Invest. 131:e145692. 10.1172/JCI145692 34263737 PMC8279590

[B9] Dong W. Chen H. Sit T. Han Y. Song F. Vyssotski A. L.. (2021). Characterization of exploratory patterns and hippocampal–prefrontal network oscillations during the emergence of free exploration. Sci. Bull. 66, 2238–2250. 10.1016/j.scib.2021.05.018 36654115

[B10] Fogaca M. V. Wu M. Li C. Li X. Y. Picciotto M. R. Duman R. S. (2021). Inhibition of GABA interneurons in the mPFC is sufficient and necessary for rapid antidepressant responses. Mol. Psychiatry 26, 3277–3291. 10.1038/s41380-020-00916-y 33070149 PMC8052382

[B11] Fonio E. Benjamini Y. Golani I. (2009). Freedom of movement and the stability of its unfolding in free exploration of mice. Proc. Nat. Acad. Sci. USA 106, 21335–21340. 10.1073/pnas.0812513106 19934049 PMC2795555

[B12] Fuchikami M. Thomas A. Liu R. Wohleb E. S. Land B. B. DiLeone R. J.. (2015). Optogenetic stimulation of infralimbic PFC reproduces ketamine's rapid and sustained antidepressant actions. Proc. Nat. Acad. Sci. USA 112, 8106–8111. 10.1073/pnas.1414728112 26056286 PMC4491758

[B13] Gilabert-Juan J. Castillo-Gomez E. Guirado R. Molto M. D. Nacher J. (2013). Chronic stress alters inhibitory networks in the medial prefrontal cortex of adult mice. Brain Struct. Funct. 218, 1591–1605. 10.1007/s00429-012-0479-1 23179864

[B14] Hare B. D. Shinohara R. Liu R. J. Pothula S. DiLeone R. J. Duman R. S. (2019). Optogenetic stimulation of medial prefrontal cortex Drd1 neurons produces rapid and long-lasting antidepressant effects. Nat. Commun. 10, 223. 10.1038/s41467-018-08168-9 30644390 PMC6333924

[B15] Higuchi F. Uchida S. Yamagata H. Abe-Higuchi N. Hobara T. Hara K.. (2016). Hippocampal microRNA-124 enhances chronic stress resilience in mice. J. Neurosci. 36, 7253. 10.1523/JNEUROSCI.0319-16.2016 27383599 PMC6705534

[B16] Hillebrand A. Tewarie P. van Dellen E. Yu M. Carbo E. W. S. Douw L.. (2016). Direction of information flow in large-scale resting-state networks is frequency-dependent. Proc. Nat. Acad. Sci. USA 113, 3867–3872. 10.1073/pnas.1515657113 27001844 PMC4833227

[B17] Hultman R. Mague S. D. Li Q. Katz B. M. Michel N. Lin L.. (2016). Dysregulation of prefrontal cortex-mediated slow-evolving limbic dynamics drives stress-induced emotional pathology. Neuron 91, 439–452. 10.1016/j.neuron.2016.05.038 27346529 PMC4986697

[B18] Jacinto L. R. Reis J. S. Dias N. S. Cerqueira J. J. Correia J. H. Sousa N. (2013). Stress affects theta activity in limbic networks and impairs novelty-induced exploration and familiarization. Front. Behav. Neurosci. 7, 127. 10.3389/fnbeh.2013.00127 24137113 PMC3797543

[B19] Kanta V. Pare D. Headley D. B. (2019). Closed-loop control of gamma oscillations in the amygdala demonstrates their role in spatial memory consolidation. Nat. Commun. 10, 3970. 10.1038/s41467-019-11938-8 31481701 PMC6722067

[B20] Likhtik E. Stujenske J. M. Topiwala M. A. Harris A. Z. Gordon J. A. (2014). Prefrontal entrainment of amygdala activity signals safety in learned fear and innate anxiety. Nat. Neurosci. 17, 106–113. 10.1038/nn.3582 24241397 PMC4035371

[B21] Liu R. J. Ota K. T. Dutheil S. Duman R. S. Aghajanian G. K. (2015). Ketamine strengthens CRF-activated amygdala inputs to basal dendrites in mpfc layer v pyramidal cells in the prelimbic but not infralimbic subregion, a key suppressor of stress responses. Neuropsychopharmacology 40, 2066–2075. 10.1038/npp.2015.70 25759300 PMC4613616

[B22] Liu W. Z. Zhang W. H. Zheng Z. H. Zou J. X. Liu X. X. Huang S. H.. (2020). Identification of a prefrontal cortex-to-amygdala pathway for chronic stress-induced anxiety. Nat. Commun. 11, 2221. 10.1038/s41467-020-15920-7 32376858 PMC7203160

[B23] Lobier M. Siebenhuhner F. Palva S. Palva J. M. (2014). Phase transfer entropy: a novel phase-based measure for directed connectivity in networks coupled by oscillatory interactions. Neuroimage 85 Pt 2, 853–872. 10.1016/j.neuroimage.2013.08.056 24007803

[B24] Ma H. Li C. Wang J. Zhang X. Li M. Zhang R.. (2021). Amygdala-hippocampal innervation modulates stress-induced depressive-like behaviors through AMPA receptors. Proc. Nat. Acad. Sci. USA 118, e2019409118. 10.1073/pnas.2019409118 33526688 PMC8017726

[B25] Marceglia S. Rossi L. Foffani G. Bianchi A. Cerutti S. Priori A. (2007). Basal ganglia local field potentials: applications in the development of new deep brain stimulation devices for movement disorders. Expert Rev. Med. Devices 4, 605–614. 10.1586/17434440.4.5.605 17850195

[B26] Marcus D. J. Bedse G. Gaulden A. D. Ryan J. D. Kondev V. Winters N. D.. (2020). Endocannabinoid signaling collapse mediates stress-induced amygdalo-cortical strengthening. Neuron 105, 1062–1076.e1066. 10.1016/j.neuron.2019.12.024 31948734 PMC7992313

[B27] McGarry L. M. Carter A. G. (2016). Inhibitory gating of basolateral amygdala inputs to the prefrontal cortex. J. Neurosci. 36, 9391. 10.1523/JNEUROSCI.0874-16.2016 27605614 PMC5013187

[B28] McGarry L. M. Carter A. G. (2017). Prefrontal cortex drives distinct projection neurons in the basolateral amygdala. Cell Rep. 21, 1426–1433. 10.1016/j.celrep.2017.10.046 29117549 PMC5714295

[B29] McNamara C. G. Rothwell M. Sharott A. (2020). Phase-dependent closed-loop modulation of neural oscillations in vivo. bioRxiv 2020.2005.2021.102335. 10.1101/2020.05.21.102335

[B30] Mitra R. Jadhav S. McEwen B. S. Vyas A. Chattarji S. (2005). Stress duration modulates the spatiotemporal patterns of spine formation in the basolateral amygdala. Proc. Nat. Acad. Sci. USA 102, 9371–9376. 10.1073/pnas.0504011102 15967994 PMC1166638

[B31] Munshi S. Rosenkranz J. A. (2018). Effects of peripheral immune challenge on in vivo firing of basolateral amygdala neurons in adult male rats. Neuroscience 390, 174–186. 10.1016/j.neuroscience.2018.08.017 30170159 PMC6168414

[B32] Ng B. S. Logothetis N. K. Kayser C. (2013). EEG phase patterns reflect the selectivity of neural firing. Cereb. Cortex 23, 389–398. 10.1093/cercor/bhs031 22345353

[B33] Pfarr S. Schaaf L. Reinert J. K. Paul E. Herrmannsdörfer F. Roßmanith M.. (2018). Choice for drug or natural reward engages largely overlapping neuronal ensembles in the infralimbic prefrontal cortex. J. Neurosci. 38, 3507. 10.1523/JNEUROSCI.0026-18.2018 29483279 PMC6596043

[B34] Qi C. Wang Z. Bai W. Liu T. Zheng X. (2020). Reduced information transmission of medial prefrontal cortex to basolateral amygdala inhibits exploratory behavior in depressed rats. Front. Neurosci. 14, 608587. 10.3389/fnins.2020.608587 33343292 PMC7744617

[B35] Rosenkranz J. A. Grace A. A. (2001). Dopamine attenuates prefrontal cortical suppression of sensory inputs to the basolateral amygdala of rats. J. Neurosci. 21, 4090. 10.1523/JNEUROSCI.21-11-04090.2001 11356897 PMC6762693

[B36] Russo S. J. Nestler E. J. (2013). The brain reward circuitry in mood disorders. Nat. Rev. Neurosci. 14, 609–625. 10.1038/nrn3381 23942470 PMC3867253

[B37] Schyns P. G. Thut G. Gross J. (2011). Cracking the code of oscillatory activity. PLoS Biol. 9, e1001064. 10.1371/journal.pbio.1001064 21610856 PMC3096604

[B38] Scott D. W. (1992). Multivariate Density Estimation. New York, NY: John Wiley & Sons, Inc.

[B39] Senn V. Wolff S. B. Herry C. Grenier F. Ehrlich I. Grundemann J.. (2014). Long-range connectivity defines behavioral specificity of amygdala neurons. Neuron 81, 428–437. 10.1016/j.neuron.2013.11.006 24462103

[B40] Stam C. J. Nolte G. Daffertshofer A. (2007). Phase lag index: assessment of functional connectivity from multi channel EEG and MEG with diminished bias from common sources. Hum. Brain Mapp. 28, 1178–1193. 10.1002/hbm.20346 17266107 PMC6871367

[B41] Tye K. M. Prakash R. Kim S. Y. Fenno L. E. Grosenick L. Zarabi H.. (2011). Amygdala circuitry mediating reversible and bidirectional control of anxiety. Nature 471, 358–362. 10.1038/nature09820 21389985 PMC3154022

[B42] Vinck M. Oostenveld R. van Wingerden M. Battaglia F. Pennartz C. M. (2011). An improved index of phase-synchronization for electrophysiological data in the presence of volume-conduction, noise and sample-size bias. Neuroimage 55, 1548–1565. 10.1016/j.neuroimage.2011.01.055 21276857

[B43] Wang Z. Cao Q. Bai W. Zheng X. Liu T. (2021). Decreased phase-amplitude coupling between the mPFC and BLA during exploratory behaviour in chronic unpredictable mild stress-induced depression model of rats. Front. Behav. Neurosci. 15, 799556. 10.3389/fnbeh.2021.799556 34975430 PMC8716490

[B44] Watrous A. J. Deuker L. Fell J. Axmacher N. (2015). Phase-amplitude coupling supports phase coding in human ECoG. Elife 4. 10.7554/eLife.07886 26308582 PMC4579288

[B45] Wei J. Zhong P. Qin L. Tan T. Yan Z. (2017). Chemicogenetic restoration of the prefrontal cortex to amygdala pathway ameliorates stress-induced deficits. Cerebral Cortex 28, 1980–1990. 10.1093/cercor/bhx104 28498919 PMC6018994

[B46] Widge A. S. Miller E. K. (2019). Targeting cognition and networks through neural oscillations: next-generation clinical brain stimulation. JAMA Psychiatry 76, 671–672. 10.1001/jamapsychiatry.2019.0740 31116372 PMC7067567

[B47] Willner P. (1997). Validity, reliability and utility of the chronic mild stress model of depression: a 10-year review and evaluation. Psychopharmacology 134, 319–329. 10.1007/s002130050456 9452163

[B48] World Health O.. (2021). Comprehensive Mental Health Action Plan 2013–2030. Geneva: World Health Organization.

[B49] Yu H. Chen L. Lei H. Pi G. Xiong R. Jiang T.. (2022). Infralimbic medial prefrontal cortex signalling to calbindin 1 positive neurons in posterior basolateral amygdala suppresses anxiety- and depression-like behaviours. Nat. Commun. 13, 5462. 10.1038/s41467-022-33139-6 36115848 PMC9482654

[B50] Zhang J. Y. Liu T. H. He Y. Pan H. Q. Zhang W. H. Yin X. P.. (2019). Chronic stress remodels synapses in an amygdala circuit-specific manner. Biol. Psychiatry 85, 189–201. 10.1016/j.biopsych.2018.06.019 30060908 PMC6747699

[B51] Zheng Z. H. Tu J. L. Li X. H. Hua Q. Liu W. Z. Liu Y.. (2021). Neuroinflammation induces anxiety- and depressive-like behavior by modulating neuronal plasticity in the basolateral amygdala. Brain Behav. Immun. 91, 505–518. 10.1016/j.bbi.2020.11.007 33161163

