# Supplementary material for: Decreased phase information transfer from the mPFC to the BLA: During exploratory behavior in CUMS rats
Source: Front Neurosci. 2023 Mar 27;17:1145721. doi: 10.3389/fnins.2023.1145721 (PMC10083315; doi:10.3389/fnins.2023.1145721)
Supplement: Supplementary file 1 [file Data_Sheet_1.docx]

Supplementary Material

**Supplementary Table 1. The number of trials in control group**

| **Subject No.** | **Number of trials** |
| --- | --- |
| **Rat1** | **26** |
| **Rat2** | **27** |
| **Rat3** | **16** |
| **Rat4** | **20** |
| **Rat5** | **15** |
| **Rat6** | **17** |
| **Total** | **121** |

**Supplementary Table 2. The number of trials in CUMS group**

| **Subject No.** | **Number of trials** |
| --- | --- |
| **Rat1** | **15** |
| **Rat2** | **18** |
| **Rat3** | **16** |
| **Rat4** | **17** |
| **Rat5** | **19** |
| **Rat6** | **14** |
| **Total** | **99** |

**Supplementary Figure legends**

**Supplementary Figure 1.** **Verification of CUMS model.** (A) Immobility time in forced swimming test. Two-tailed Mann Whitney test, 31.887±2.653, n=6 rats for control vs. 92.733±2.807, n=6 rats for CUMS, **P<0.01. (B) Sucrose preference rate in sucrose preference test. Two-tailed Mann Whitney test, 88.45±1.528, n=6 rats for control vs. 54.348±3.798, n=6 rats for CUMS, **P<0.01.

**Supplementary Figure 2.** **The mean WPLI time-frequency heat map of each rat.** (A) Spectrograms of mean WPLI between mPFC and BLA during exploration in control rats. (B) Spectrograms of mean WPLI between mPFC and BLA during exploration in CUMS rats. n=6 rats per group. Dashed line refers to reference point.

**Supplementary Figure 3.** **WPLI at different frequency bands within mPFC-BLA in the control and CUMS group.** Error bars indicate SEM. Two-way repeated measures ANOVA, ns, P>0.05, not significant.

**Supplementary Figure 4.** **The mean PTE (from mPFC to BLA) curve with time in theta-range of each rat.** (A) Plots of PTE from mPFC to BLA during exploration in control rats. (B) Plots of PTE from mPFC to BLA during exploration in CUMS rats. n=6 rats 121 trials for control group, n=6 rats 99 trials for CUMS group. Faded bands, ±SEM.

**Supplementary Figure 5.** **Data verification.** (A) Example LFPs data during exploration (-2s - 2s) in the mPFC (top) and BLA (bottom), respectively. (B) Representative PTE (from the mPFC to BLA) curve with time during one exploration. (C) Representative comparison diagram of actual and shuffled PTE (from the mPFC to BLA) from one trial. Blue curve, averaged PTE of 500 × shuffled controls; faded bands, ±SEM. Yellow curve, actual PTE. (D) The histogram of peak PTE of shuffled controls. Sapphire blue line indicates the threshold of peak PTE of shuffled controls, it was set at 0.95 percentile of the mean peak PTE of randomized controls. Yellow line indicates the actual peak PTE during one behavior. The representative result shows that actual peak PTE is significantly higher than values obtained from shuffled controls.
