# Supplementary figures and images for: Decreased phase information transfer from the mPFC to the BLA: During exploratory behavior in CUMS rats
Source: Front Neurosci. 2023 Mar 27;17:1145721. doi: 10.3389/fnins.2023.1145721 (PMC10083315; doi:10.3389/fnins.2023.1145721)

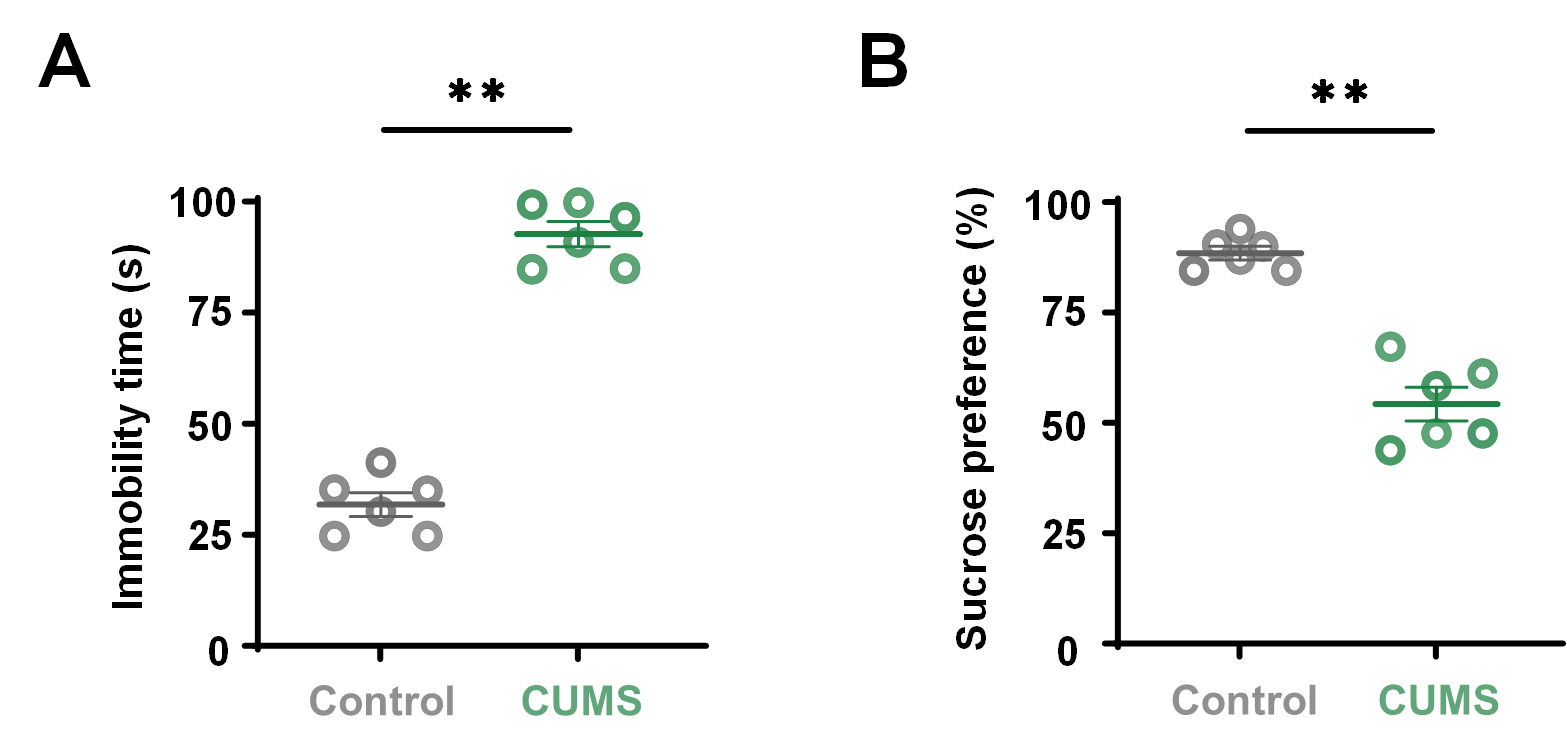

Supplement: Supplementary file 2 [file Image_1.TIF]

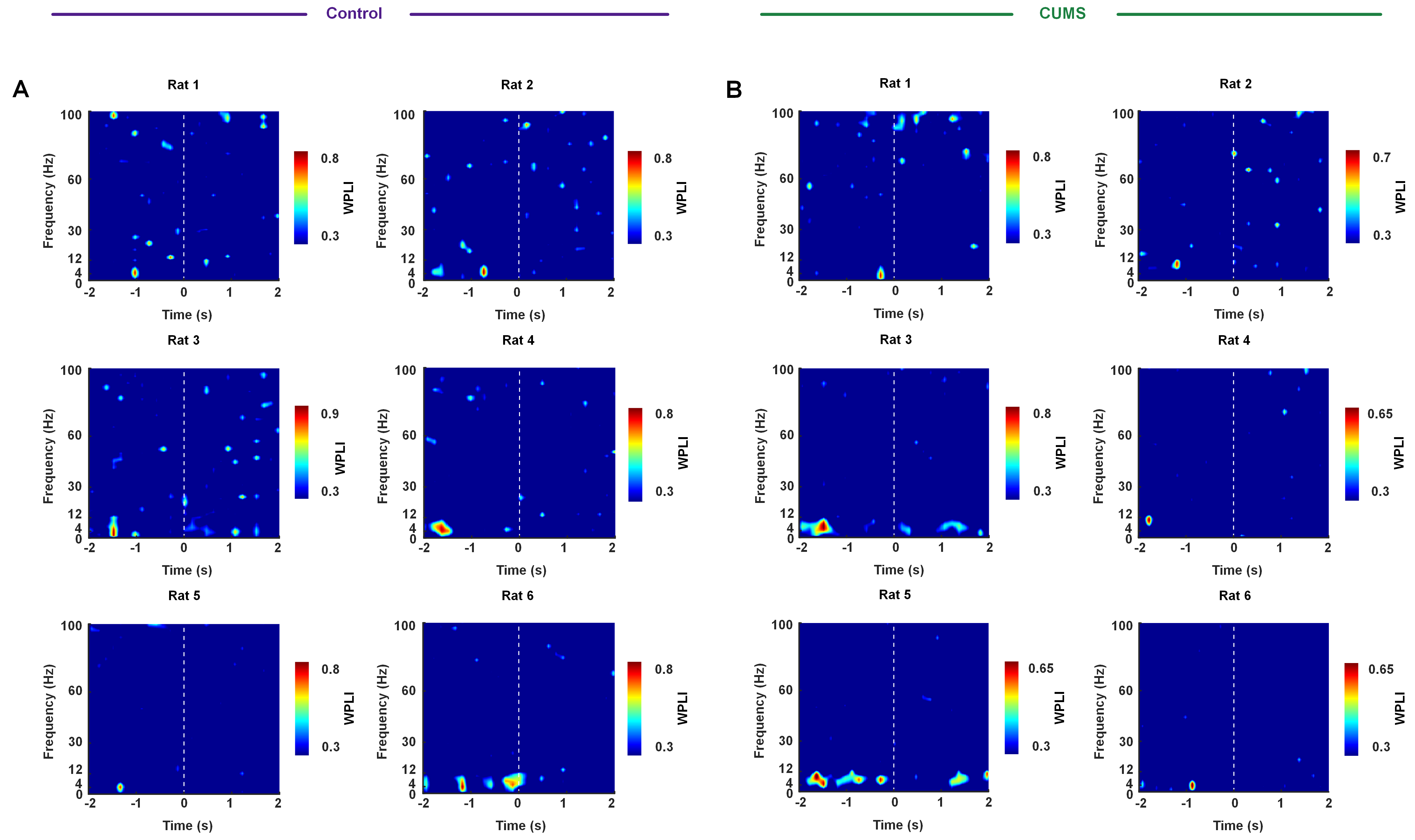

Supplement: Supplementary file 3 [file Image_2.TIF]

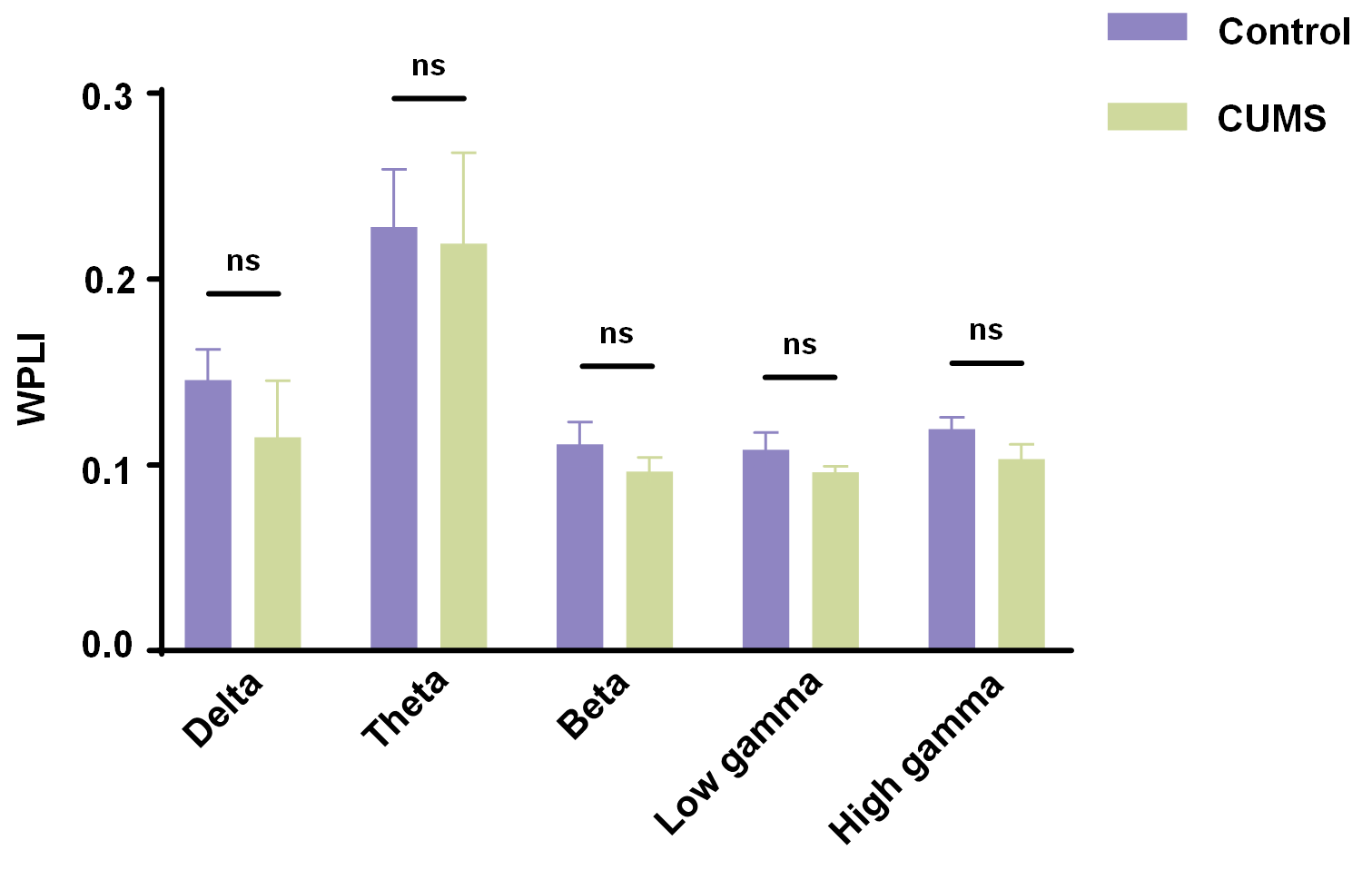

Supplement: Supplementary file 4 [file Image_3.TIF]

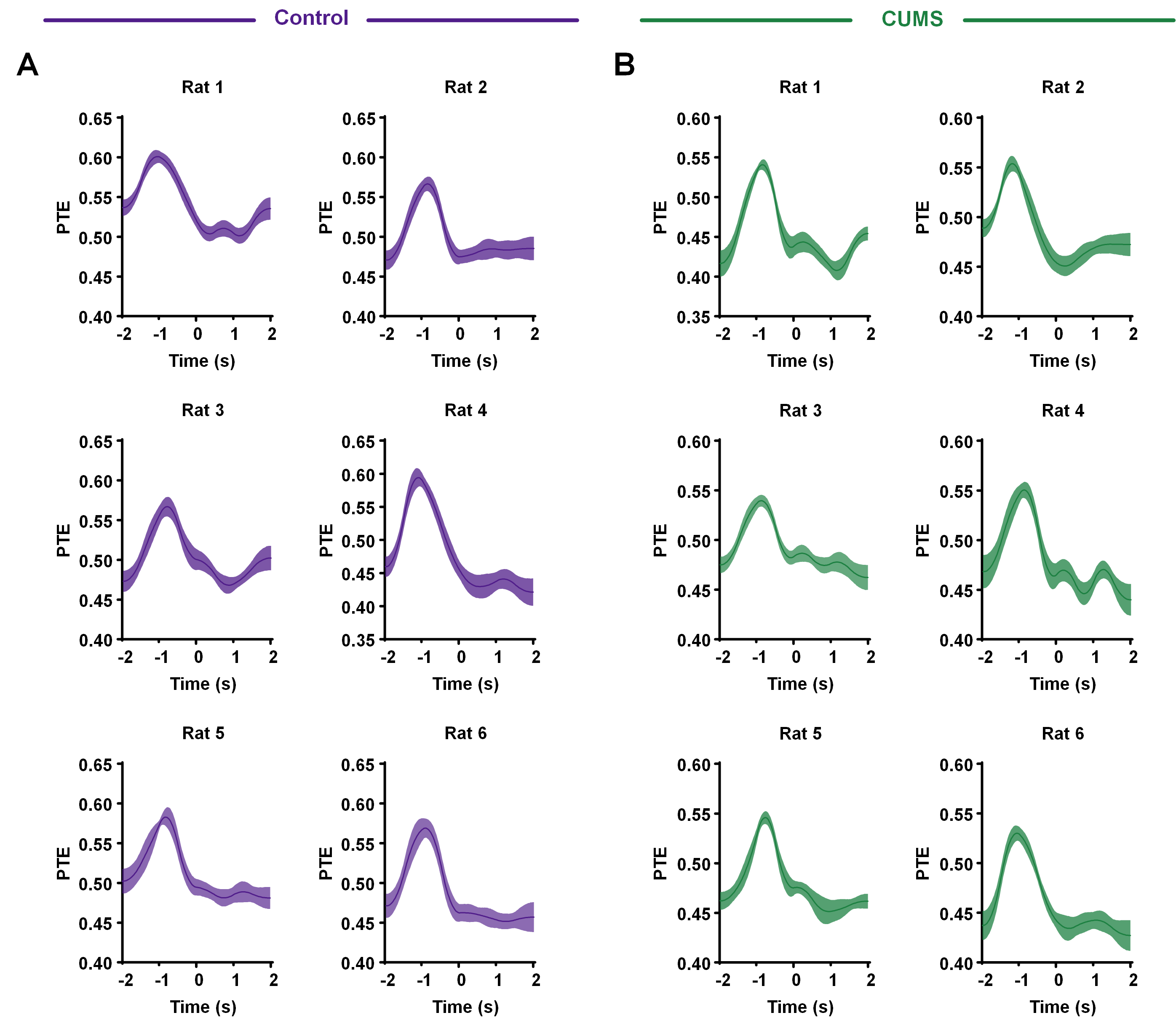

Supplement: Supplementary file 5 [file Image_4.TIF]

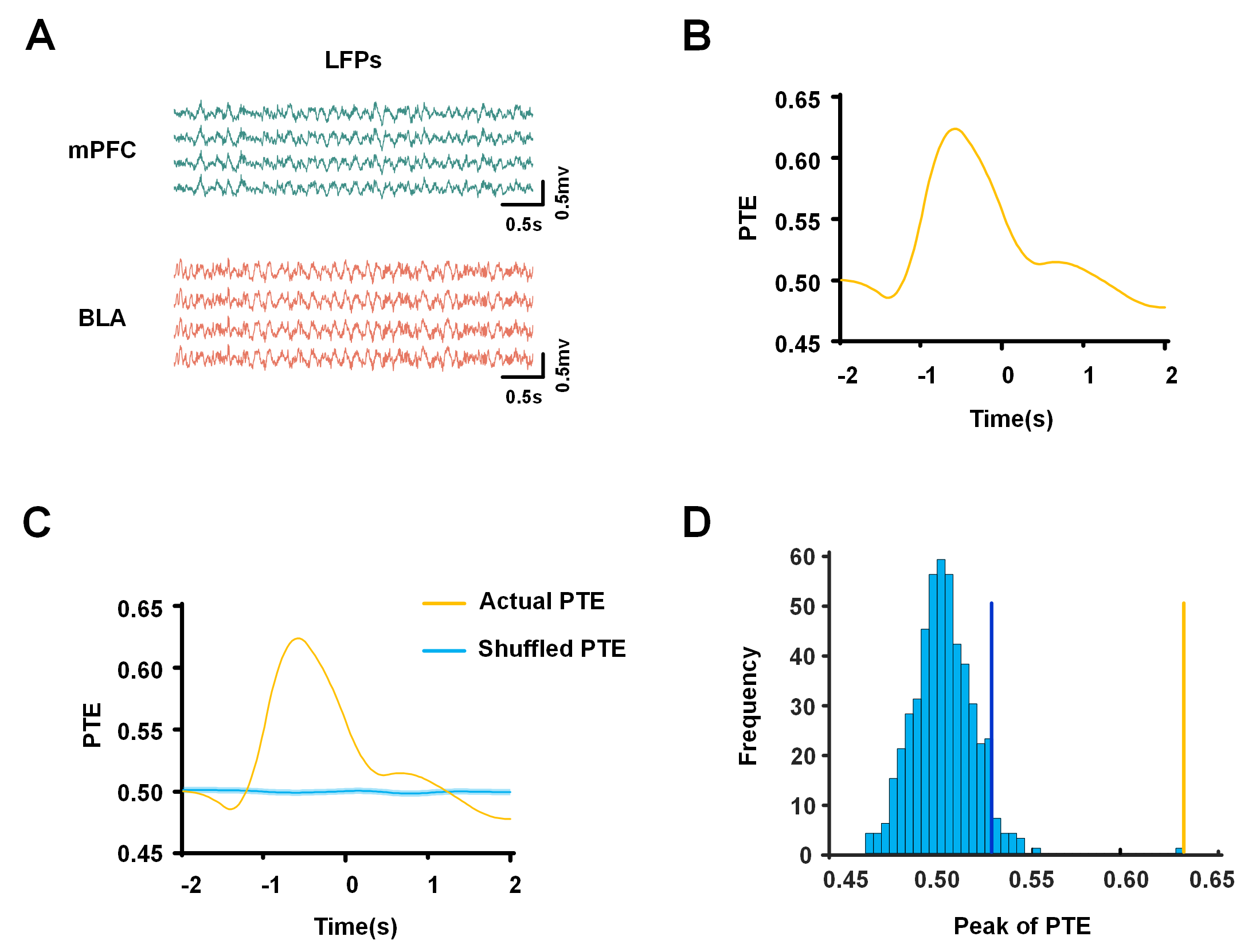

Supplement: Supplementary file 6 [file Image_5.TIF]
